# Can self-rated health be useful to primary care physicians as a diagnostic indicator of metabolic dysregulations amongst patients with type 2 diabetes? A population-based study

**DOI:** 10.1186/s12875-024-02671-3

**Published:** 2025-05-16

**Authors:** K. Umeh, S. Adaji

**Affiliations:** 1 School of Psychology, Faculty of Health, Liverpool John Moores University, Liverpool, Merseyside L3 3AF UK; 2 Sessional General Practitioner, Bousfield Health Centre, Westminster Road, Liverpool, L4 4PP UK

**Keywords:** Diabetes, Metabolic syndrome, Self-perception, Cardiometabolic risk factors

## Abstract

**Background:**

Although most of the management of type 2 diabetes (T2DM) occurs in primary care, and physicians are tasked with using a ‘whole person’ approach, there is currently a lack of research on psychosocial diagnostic indicators for detecting metabolic abnormalities in T2DM patients. This study examined relations between SRH and metabolic abnormalities in patients with type 2 diabetes, adjusting for metabolic comorbidity.

**Method:**

A total of 583 adults with type 2 diabetes were identified from the 2019 HSE (Health Survey for England). Data on metabolic syndrome (MetS) was extracted, including lipids (high density lipoprotein cholesterol (HDL-C)), glycated haemoglobin (HbA1c), blood pressure (systolic/diastolic), and anthropometric measures (BMI, waist/hip ratio). Bootstrapped hierarchical regression and structural equation modelling (SEM) were used to analyse the data.

**Results:**

Adjusting for metabolic covariates attenuated significant associations between SRH and metabolic abnormalities (HDL-C, HbA1c), regardless of MetS status. Analysis by gender uncovered covariate-adjusted associations between SRH and both HDL-C (in men) and HbA1c (in women) (*p*’s = 0.01), albeit these associations were no longer significant when evaluated against a Bonferroni-adjusted alpha value (*p* > 0.004). Sensitivity analysis indicated most findings were unaffected by the type of algorithm used to manage missing data. SEM revealed no indirect associations between SRH, metabolic abnormalities, and lifestyle factors.

**Conclusions:**

While poor SRH can help primary care physicians identify T2DM patients with metabolic dysfunction, it may not offer added diagnostic usefulness over clinical biomarkers.

## Background

### Primary care

**Fig. 1:**
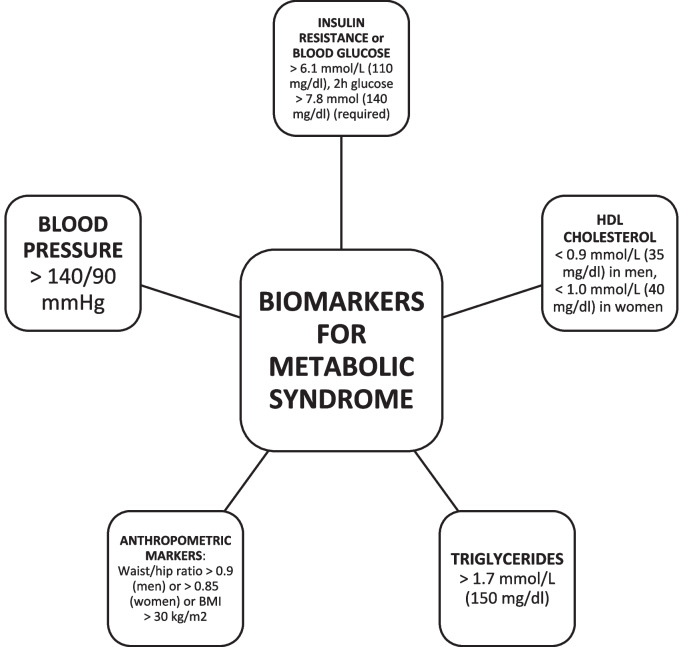
Diagnostic criteria for metabolic syndrome based on WHO (1999) guidelines (Source: Saklayen, [2])

Most of the management of type 2 diabetes (T2DM) occurs in primary care [1]. Primary care physicians are expected to adopt a ‘whole-person’ (holistic) approach, including bio-psycho-social evaluations, when working with patients to detect and manage metabolic abnormalities that increase the risk of cardiovascular disease, and other cardiometabolic complications [2], such as insulin resistance, elevated fasting glucose (≥ 100 mg/dL), waist circumference (> 0.9 (men) or > 0.85 (women)), triglycerides (≥ 150 mg/dL (1.7 mmol/L), blood pressure (systolic ≥ 130 and/or diastolic ≥ 85 mm Hg), and reduced HDL-C (< 40 mg/dL (1.0 mmol/L) in males; < 50 mg/dL (1.3 mmol/L) in females) (see Fig. 1) [3]. The presence of insulin resistance or elevated fasting glucose, and any two of the aforementioned criteria, is considered diagnostic of metabolic syndrome (MetS) [2].

While MetS is especially problematic in people with T2DM [4], metabolic irregularities often do not produce overt symptoms (besides visible abdominal adiposity in some patients) [5]. This can be problematic in primary care settings, where the focus is on identifying and reducing metabolic abnormalities [1]. Clinicians need to conduct a thorough physical examination to diagnose the condition [6]. Despite the growing emphasis on a biopsychosocial approach in the management of T2DM in primary care settings [7], there has been limited research on psychological diagnostic indicators that primary care physicians can use to detect metabolic dysregulations in asymptomatic T2DM patients.

### Self-rated health

Self-rated health (SRH) is an increasingly important construct in epidemiological and biomedical research [8–10]. It refers to a person’s assessment of their health status and is thought to be a more accurate health indicator than biomedical risk factors [11]. For example, SRH may depict undiagnosed illness at preclinical or prodromal stages (i.e., before major symptoms appear) [8]. It is a simple and easy to administer measure and hence can be a useful risk indicator in clinical settings (e.g., during doctor-patient consultations). Decades of research suggests SRH is a reliable predictor of mortality, over and beyond physical health indicators, with its predictive power increasing over time [9]. Research also suggests SRH independently predicts morbidity, including cardio cerebral vascular diseases, after adjusting for biomedical and sociodemographic covariates [12–15].

Recently, there has been growing interest in the relationship between SRH and metabolic health [16–18], notably the specific metabolic abnormalities used to define MetS, such as insulin resistance, hyperlipidaemia (high cholesterol), blood pressure, and anthropometric factors [19–21]. An association between SRH and metabolic function may be underpinned by several mechanisms. First, a person may simply perceive *symptoms* of metabolic dysfunction (e.g., weight gain), and consequently infer that they are in a poor state of health [8]. This scenario assumes that illness symptoms are perceptible (i.e., the person is not asymptomatic) [22, 23]. Second, an individual may evaluate their health status based on *biomarker* information depicting metabolic functioning, such as clinical test results, or data from medical tests performed at home (e.g., blood pressure monitoring) [10]. Third, SRH may reflect the presence of various *risk factors* for metabolic dysfunction, including family history, behavioural risk factors, and/or or signs of declining health, such as functional impairment [8].

### Ambiguity in the literature

Historically, previous research demonstrating associations between SRH and MetS have rarely controlled for the specific clinical biomarkers that define MetS [21]. SRH has been linked to various metabolic abnormalities including high density lipoprotein cholesterol (HDL-C) [24, 25], triglycerides [20], and blood pressure [26–28]. While some studies have adjusted for anthropometric markers, notably BMI [20], we found no study controlling for other metabolic dysfunctions in MetS (e.g., HDL-C, triglycerides, blood glucose, systolic/diastolic blood pressure). Thus, it remains unclear how associations between SRH and metabolic abnormalities is affected by related metabolic factors.

This problem is well illustrated in a large-scale investigation using data from three European populations (approximately 15,000 individuals). The study found that SRH was associated with at least 57 (out of 150) biomarkers, including biochemical factors that define MetS, such as HDL-C (mmol/L), triglycerides (mg/dl) glycaeted haemoglobin (HbA1c, %), and insulin (mU/ml) [10]. Although these associations were independent of disease and physical functioning (e.g., number of diseases), there was no adjustment for metabolic covariates. This methodological constraint was also manifest in another large-scale population-based study using data from 18,000 adults [13]. Although SRH was found to be associated with metabolic anomalies such as haemoglobin, triglycerides, LDL-C (low-density lipoprotein cholesterol), and fasting plasma glucose, the study did not adjust for covariance between these metabolic biomarkers.

The ambiguity in the SRH literature is problematic since biomedical research indicates significant multimorbidity in metabolic biomarkers [29–31]. For example, consider a scenario in which poor SRH depicts a specific aspect of hyperlipidemia, such as HDL-C deficiency [32]. SRH may simply be capturing comorbid cardiometabolic abnormalities that primary care physicians can easily observe, and/or detect using available clinical options (e.g., obesity, HbA1c) [10]. In this scenario, SRH does not provide primary care practitioners with any unique insights in detecting and managing cardiometabolic complications in T2DM patients. Consequently, in order to show that SRH offers unique diagnostic utility for detecting metabolic dysfunction in T2DM patients, over and beyond comorbid biomarkers [10], it is necessary to adjust for cardiometabolic covariates.

### Asymptomatic patients

Although research has implicated SRH in cardiometabolic health amongst patients with T2DM [33, 34], evidence is limited, and it remains unclear how SRH contributes to metabolic abnormalities in this clinical population. Not every T2DM patient meets the criteria for MetS [2]. Contrary to the prevailing pathophysiological perspective that metabolic dysfunction applies to all T2DM cases, a cross-sectional analysis of 414 T2DM cases (including body weight and fat mass, systolic/diastolic blood pressure, and glucose tolerance) found that 15% displayed no components of MetS, other than hyperglycaemia [35]. Although these cases showed insulin resistance, other metabolic levels (e.g., triglycerides, HDL-C, and blood pressure) matched concentrations in healthy controls. Certain forms of metabolic dysregulation do not generate any symptoms (e.g., high cholesterol), meaning clinicians need to conduct thorough physical examinations and blood testing to diagnose the condition [6]. Thus, a significant relationship between SRH and metabolic abnormalities, independent of other metabolic biomarkers, will be clinically relevant to T2DM patients, since poor SRH may help identify asymptomatic patients with subclinical metabolic dysfunctions, before the development of overt clinical MetS [8]. SRH is an easily measured metric [11], and hence may be especially useful in clinical settings by providing doctors with an extra diagnostic tool to identify high risk T2DM patients requiring additional clinical evaluation, to detect metabolic anomalies.

### Research objectives

Professionals in primary care settings face a growing plethora of available clinical options for detecting and managing metabolic abnormalities in T2DM [1]. However, despite the emphasis on a holistic approach in primary care [7], there has been limited research on useful psychological diagnostic indicators for detecting metabolic dysregulations in T2DM patients. While it is possible SRH may be a useful diagnostic indicator for detecting asymptomatic metabolic dysfunction in T2DM patients, currently there has been little or no research testing this premise. Although past studies have demonstrated significant associations between SRH and metabolic anomalies [16, 18–21], independent of disease and physical functioning [10], these relationships may be confounded by comorbid metabolic biomarkers [29, 30]. Thus, it is necessary to demonstrate extent to which SRH depicts metabolic abnormalities in T2DM patients, while accounting for cardiometabolic covariates [1].

The current study examined two specific questions:

- Does SRH *independently* predict metabolic abnormalities in T2DM patients? Consistent with previous research on SRH in relation to biomarkers [10], and MetS [20], we expected independent associations between SRH and metabolic variables after adjusting for metabolic covariates (Hypothesis 1).
- Does SRH *independently* predict metabolic abnormalities differentially in T2DM patients who do and those who do not meet MetS diagnostic criteria? Based on research linking SRH to biomarkers, independent of disease diagnosis [10], we hypothesised independent associations between SRH and metabolic factors after adjusting for metabolic covariance, irrespective of MetS status (Hypothesis 2) [8, 36, 37].

## Materials & methods

### Ethics statements

The study was conducted according to the guidelines of the Declaration of Helsinki and approved by the Institutional Review Board (or Ethics Committee) of Liverpool John Moores University, covering research with archived data from the Health Survey for England (HSE) (approval number 16/NSP/035, 14 June 2016).

### Data availability

The Health Survey for England (HSE) is managed by the National Centre for Social Research (NatCen) and the Department of Epidemiology and Public Health at University College London. HSE data cannot be shared publicly for legal and ethical reasons, third party rights, and institutional or national regulations or laws. The UK Data Service provides restricted access to HSE data, to protect confidential or proprietary information. Individuals and organisations seeking access need to be registered with the UK Data Service, albeit access is limited to applicants from UK HE/FE institutions, central and local government, NHS, research companies and charities for not-for-profit education and research purposes. Users not in the above categories can submit access requests to surveys.queries@nhs.net and will be subject to approval. For more information, please contact the UK Data Service website. https://rb.gy/vhi5uf.

### Design

**Fig. 2:**
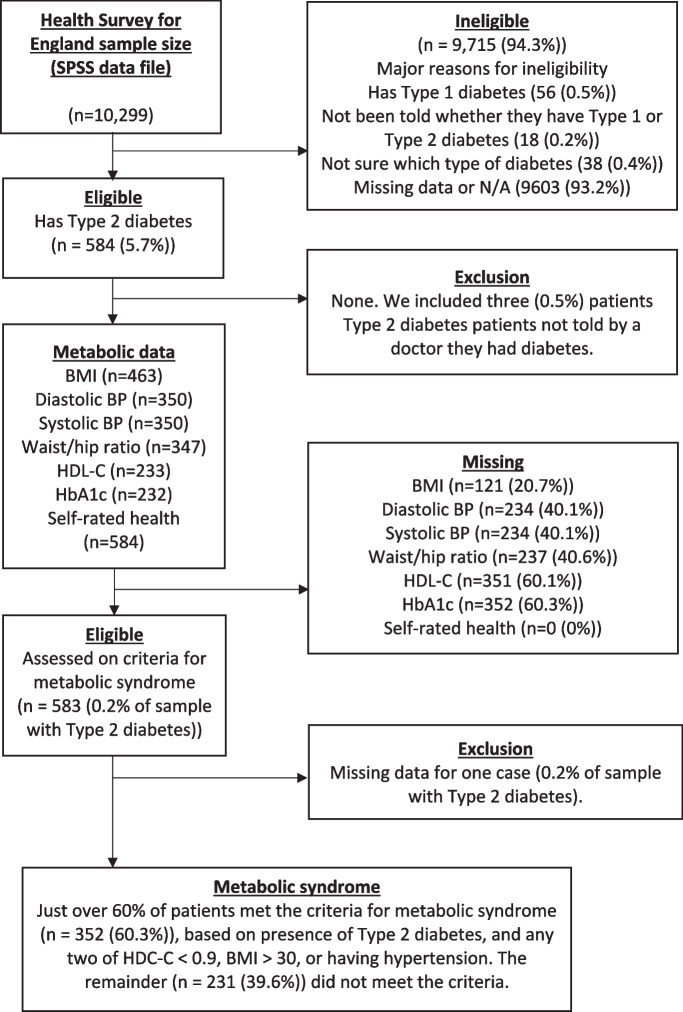
Flow Diagram

Figure 2 shows participant recruitment and eligibility data. We extracted data from the 2019 Health Survey for England (HSE), which monitors health-related trends in adults (aged > 16) and children (aged 0 to 15) living in England, United Kingdom [38]. The HSE is conducted by the National Centre for Social Research (NatCen) and the Department of Epidemiology and Public Health at University College London. HSE data cannot be shared publicly for legal and ethical reasons, due to third party rights, institutional or national regulations or laws, and the nature of data gathered. Access to HSE data is provided by the UK Data Service under restrictions to protect confidential or proprietary information. The survey assesses various biomedical parameters, including metabolic risk factors (e.g., height, weight, blood pressure, lipid profiles), lifestyle (e.g., smoking and alcohol use) and SRH. In general, survey protocol involves an interview and/or completion of a questionnaire followed by a visit from a nurse who collects biomedical data including saliva samples. Details of 2019 HSE methodology and scope, including the questionnaire, have been published elsewhere [39].

### Sample

A total of 8,205 adults and 2,095 children (total = 10,300) participated in the 2019 survey. Of these, 4,947 adults and 1,169 children were visited by a nurse. Participants were recruited using stratified probability sampling, to ensure the sample is representative of the household population in England. Only participants diagnosed with T2DM by a doctor or nurse were eligible to participate in the present study. We identified 584 individuals with T2DM, of whom 353 (60.4%) met the diagnostic criteria for MetS.

### Self-rated health

SRH data was assessed via the question “How is your health in general? Would you say it was …” (respondents selected one of five responses options: “Very good” (coded 1), “Good” (coded 2), “Fair” (coded 3) “Bad” (coded 4), and “Very bad” (coded 5)). These response options differ from categories used in some other research, which for example include an “excellent” option [10]). For linear regression SRH was collapsed into a simple dichotomous (dummy) variable due to the very small number of MetS cases in the “Very good” (n = 27) and “Very bad” (n = 27) categories. For this new variable “fair”/”bad”/”very bad” responses were coded 0, while “good”/”very good” responses were coded 1. For the purposes of conducting structural equation modelling (SEM) with maximum likelihood estimation (which requires continuous data), SRH was treated as continuous variable with the five original categories (recoded from 0 (“Very good”) through to 4 (“Very bad”)). Thus, a higher value indicated poorer SRH.

### Metabolic variables

Metabolic data was based on blood samples taken during the nurse visit [38]. All measures were treated as both continuous variables (for regression analysis) and dichotomised variables, based on MetS diagnostic criteria, in order to identify MetS cases [2]. Serum HDL-C was measured in mmol/L, with 0.9 mmol/L (35 mg/dl) for men used as the critical threshold (≥ 0.9 mmol/L (coded 0) vs. < 0.9 mmol/L (coded 1)). Anthropometric markers consisted of waist/hip ratio data, with 0.85 (women) used as the critical threshold (> 0.85 (coded 1) vs. < 0.85 (coded 0)) and BMI scores, dichotomised based on the cut-off for obesity (> 30 kg/m^2^ (coded 1) vs. < 30 kg/m^2^ (coded 0)). Diagnosis with hypertension by a health professional was a simple dichotomy (‘Yes’ (coded 1) vs. ‘No’ (coded 0)). We also extracted systolic and diastolic blood pressure data, viewed as separate biomarkers due to differential effects on health outcomes [40]. Both variables were dichotomised: systolic (≤ 120 mm Hg (coded 0) vs. > 120 mm Hg (coded 1)); diastolic (≤ 80 mm Hg (coded 0) vs. > 80 mm Hg (coded 1)). Finally, we extracted glycaeted haemoglobin (HbA1c (mmol/mol)) data, in place of fasting glucose. Inclusion of HbA1c here reflects the new clinical definition for MetS proposed by the IDF (International Diabetes Federation), [41]. HbA1c scores were dichotomised at the 48 mmol/mol clinical threshold for diabetes; < 48 mmol/mol (coded 0) or =  > 48 mmol/mol (coded 1) [42].

WHO criteria were used to identify MetS cases [5]. This entails insulin resistance or glucose > 6.1 mmol/L (110 mg/dl), 2 h glucose > 7.8 mmol (140 mg/dl), and any two of four additional diagnostic requirements: (a) serum HDL-C (cholesterol) < 0.9 mmol/L (35 mg/dl) for men, and < 1.0 mmol/L (40 mg/dl) for women, (b) triglycerides > 1.7 mmol/L (150 mg/dl), (c) a waist/hip ratio > 0.9 for men, or > 0.85 for women, or a BMI value > 30 kg/m2, and (d) blood pressure > 140/90 mmHg. Since data on insulin resistance and impaired glucose tolerance was unavailable [39], we assumed poor insulin sensitivity from T2DM status [43]. Furthermore, BMI (> 30 kg/m^2^) rather than waist/hip ratio was used as the primary anthropometric measure since the former criterion is not gender-specific [44]. We also applied the HDL-C threshold for men (< 0.9 mmol/L (35 mg/dl)) as this is more conservative. Additionally, diagnosis with hypertension was used in place of systolic/diastolic blood pressure readings, due to the greater proportion of missing data for the latter. Overall, MetS caseness was based on the presence of T2DM and any two of the following: serum HDL-C < 0.9 mmol/L (35 mg/dl); BMI (kg/m^2^) > 30; diagnosis with hypertension by a health professional. A total of 352 MetS cases (60.3%) were identified using these criteria (MetS cases = 1, non-cases = 0).

### Other covariates

We assessed two lifestyle factors: cigarette smoking and alcohol consumption. Both behaviours are heavily implicated in MetS and increased cardiovascular risk [45, 46]. For example, a population-based study of 64,046 adults (aged 18 to 80) found MetS prevalence varied as a function of both smoking and alcohol consumption. Current alcohol and cigarette use predicted higher cholesterol (triglycerides) levels, and alcohol intake was linked to truncal obesity and increased blood pressure, with the latter effect more pronounced in heavy smokers [47]. We extracted two lifestyle items from the HSE data, each treated as a single-item measure: one assessed number of cigarette smoked per day (respondents provided a numerical figure), while the other assessed the frequency of alcohol consumption in the past twelve months: respondents selected one of eight categories (“Almost every day” (coded 1), “Five or six days a week” (coded 2), “Three or four days a week” (coded 3), “Once or twice a week” (coded 4), “Once or twice a month” (coded 5), “Once every couple of months” (coded 6), “Once or twice a year” (coded 7), and “Not at all in the last 12 months” (coded 8)). Both lifestyle measures were treated as quantitative variables, with a higher score denoting higher levels of cigarette use or alcohol consumption.

We extracted data for four demographic factors: age, gender, socio-economic status, educational level, and ethnicity. Age was calibrated in twenty-two bands: ages 1 to 16 were classified into six 1- or 2-year age bands (e.g., 2–4, 13–15), while ages over 16 were grouped into 3- or 4-year age bands (e.g., 16–19, 30–34, 75–70). Gender was a dichotomy: male (coded 1), female (coded 0). Socio-economic classification contained eight bands using the UK Registrar General’s scale: (code = 0) ‘higher managerial and professional’, (code = 1) ‘lower managerial and professional’, (code = 2) ‘intermediate occupations’, (code = 3) ‘small employers & own account workers’, (code = 4) ‘lower supervisory and technical’, (code = 5) ‘semi-routine occupations’, (code = 6) ‘routine occupations’, and (code = 7) ‘never worked & long-term unemployed’. Level of educational level was dichotomised: ‘below degree or none’ (coded 0) and ‘degree or equivalent’ (coded 1). Finally, ethnicity was also a simple dichotomy: ‘White’ (coded 0) and ‘non-White’ (coded 1).

### Data analysis

We performed chi-square and independent samples t-tests to evaluate group differences in metabolic function based on MetS status. Bootstrapped hierarchical multiple regression was used to test each hypothesis. In each regression analysis we predicted an individual metabolic variable (e.g., HDL-C), with all other metabolic factors treated as covariates. We constructed three models for each regression analysis: Model 1 (metabolic variable = Intercept + Age + Gender + Social Class + Ethnicity + Lifestyle factors), Model 2 (metabolic variable = Intercept + Age + Gender + Social Class + Ethnicity + Lifestyle factors + SRH), Model 3 (metabolic variable = Intercept + Age + Gender + Social Class + Ethnicity + Lifestyle factors + SRH + other metabolic factors). Thus, metabolic covariates were included in the equation after first evaluating the predictive utility of SRH. We initially adopted a lower alpha level (*p* ≤ 0.01), to reduce type 1 errors, but interpreted significant regression results using a more conservative Bonferroni-adjusted alpha value (*p* < 0.004), to further reduce the risk of false positives [48]. Power analysis for multiple regression using G*Power 3.1.7 [49] indicated a minimum total sample size of N = 234, to detect a medium effect (f^2^ = 0.15), at a 0.01 alpha level, and 95% power (1 – β err prob) [50].

## Results

### Descriptive statistics

We employed listwise deletion to manage missing data [51], which ranged from 0% for demographics (age, gender, ethnicity) to > 20% for BMI, and > 40% for diastolic/systolic blood pressure (40.1% each), and waist/hip ratio (40.6%), to as high as 60% for education level (61%), HbA1c (60.3%), and HDL-C (60.1%) (see Fig. 2). Despite the limitations of listwise deletion, this approach was preferred to inputting (replacing) missing data using estimated parameters (e.g., expectation maximisation). The latter methods require assumptions of multivariate normality, which is problematic with categorical variables (e.g., SRH, MetS) [52]. Regardless, we performed sensitivity analysis to compare the effects of listwise deletion versus expectation maximisation on regression results.

Of 584 patients diagnosed with T2DM, 353 patients (60.3%) met the criteria for MetS. It should be noted that occurrence of MetS in diabetes patients varies, and may be influenced by various factors including MetS diagnostic criteria: thus not every diabetes patient is diagnosed with MetS [53]. The percentage of patients meeting each individual diagnostic criterion are as follows: HDL-C <  = 0.9 mmol/L (35 mg/dl) (n = 391 (67%)), waist/hip ratio =  > 0.85 cm (n = 316 (54.1%)); BMI > 30 kg/m2 (n = 229 (39.2%)); diagnosed with hypertension by a doctor or nurse; (n = 370 (63.4%)): systolic blood pressure > 140 mmHg (n = 82 (14%)) and diastolic blood pressure > 90 mmHg (n = 14 (2.4%)). Just over a quarter of patients had a HbA1c > 48 mmol/mol (n = 167 (28.6%)). The percentage of participants per SRH category were ‘very good’ (9.8%), ‘good’ (32.9%), ‘fair’ (34.8%), ‘bad’ (16.1%), and ‘very bad’ (6.5%). Thus, just over 40% of patients reported ‘good’/’very good’ health.

Table 1 shows means, SDs, and frequencies for the overall sample and by MetS status (cases versus non-cases). All participants were aged ≥ 16 years, with most participants (56.8%) aged ≥ 65 years. The youngest age band was 16 to 19 years, the oldest was 90 + years, while the median age band was 65 to 69 years. The sample was predominantly male (54.1%), 486 (83.2%) identified as Caucasian, 105 (47.1%) had a university education at degree level or equivalent, and 184 (33%) came from the top three socio-economic groups (higher/lower managerial, professional, intermediate occupations).

**Table 1:** Sample characteristics by metabolic syndrome status

	Whole sample	Metabolic syndrome		*P*
		Non-cases	Cases	
Age, n (%) ≥ 65 years	332 (56.8%)	123 (53.2%)	208 (59.1%)	*P* > 0.01
Gender, n (%) male	316 (54.1%)	123 (53.2%)	193 (54.8%)	*P* > 0.01
Socio-economic class, n (%) managerial, professional, intermediate	184 (32.9%), missing 25 (4.3%)	77 (35.6%)	107 (31.4%)	*P* > 0.01
Ethnicity, n (%) White	486 (83.2%)	183 (79.2%)	302 (85.8%)	*P* > 0.01
Education, n (%) university/college degree or equivalent	105 (18%), missing 361 (61.8%)	48 (48%)	57 (46.3%)	*P* > 0.01
Cigarette smoking (number of cigarettes smoked a day)	2.28 (7.10)	2.25 (6.59)	2.31 (7.44)	*P* > 0.01
Alcohol consumption frequency in past year, n (%) not at all/non-drinker	183 (31.3%), missing 1 (0.2%)	72 (31.3%)	111 (31.5%)	*P* > 0.01
Self-rated health, n (%) ‘fair’/ ‘bad’/ ‘very bad’ health	335 (57.4%)	111 (48.1%)	223 (63.4%)	*P* < 0.01*
HDL-C (mmol/L), n (%) ≤ 0.9	391 (67%)	101 (43.7%)	289 (82.1%)	*P* < 0.01*
HDL-C (mmol/L)	1.25 (0.33)	1.30 (0.32)	1.18 (0.33)	*P* < 0.01*
Waist/hip ratio (cm), n (%) ≥ 0.85	316 (54.1%)	146 (63.2%)	169 (48%)	*P* < 0.01*
Waist/hip ratio (cm)	0.96 (0.08)	0.94 (0.07)	0.98 (0.08)	*P* < 0.01*
BMI, n (%) ≥ 30 kg/m^2^	229 (39.2%)	18 (7.8%)	211 (59.9%)	*P* < 0.01*
BMI kg/m^2^	31.22 (6.11)	27.54 (3.63)	33.41 (6.25)	*P* < 0.01*
Systolic blood pressure, n (%) > 140 mmHg	82 (14%)	38 (16.5%)	43 (12.2%)	*P* > 0.01
Systolic blood pressure, mmHg	129 (16.18)	128.49 (15.96)	129.34 (16.37)	*P* > 0.01
Diastolic blood pressure, n (%) > 90 mmHg	14 (2.4%)	6 (2.6%)	8 (2.3%)	*P* > 0.01
Diastolic blood pressure, mmHg	69.72 (10.53)	69.46 (10.23)	69.99 (10.84)	*P* > 0.01
Hypertension (diagnosed)	370 (63.4%)	60 (26%)	310 (88.1%)	*P* < 0.01*
HbA1c, n (%) > 48 mmol/mol	167 (28.6%)	102 (44.2%)	65 (18.5%)	*P* < 0.01*
HbA1c, mmol/mol	57.5 (16.56)	56.17 (15.05)	59.61 (18.60)	*P* > 0.01

All values are means (SDs), unless percentage (%) stated. P values relate to comparisons between metabolic syndrome cases versus non-cases, are based on Chi-square or independent samples t-tests (* indicates significant)

Respondents smoked an average of 2.28 cigarettes a day, and consumed alcohol 5.6 times in the past 12 months. The sample met WHO thresholds for obesity (BMI (kg/m2) > 0.30 (M = 31.22)), high central adiposity (waist/hip ratio (cm) > 0.9 (men) (M = 1.00), > 0.85 (women) (M = 0.91)), and poor glycaemic control (HbA1c > 48 mmol/mol) (M = 57.50). HDL-C levels were normal (i.e., above minimum thresholds of < 0.9 mmol/L in men (M = 1.19) and < 1.0 mmol/L in women (M = 1.31)). Systolic/diastolic blood pressure values were also below the critical thresholds of > 140/90 mmHg (M = 129/69.72).

MetS cases were significantly less likely to report ‘very good’/ ‘good’ SRH (χ2 (1, N = 583) = 13.344, *p* < 0.001). There were no group differences in demographic factors or systolic/diastolic blood pressure (all *p*’s > 0.01), albeit a slightly higher proportion of MetS cases (59.1%) were aged 65 years or older, compared with non-cases (53.2%). MetS cases were significantly more likely than non-cases to be HDL-C deficient (HDL-C <  = 0.9 mmol/L (35 mg/dl)) (χ2 (1, N = 583) = 92.768, *p* < 0.001), and generally overweight (BMI > 30 kg/m2), (χ2 (1, N = 583) = 159.041, *p* < 0.001), but less likely to be centrally obese (waist/hip ratio =  > 0.85 cm), (χ2 (1, N = 583) = 12.960, *p* < 0.001). MetS cases were also more likely to be hypertensive (χ2 (1, N = 583) = 231.923, *p* < 0.001), but show better glycaemic control (HbA1c > 48 mmol/mol), (χ2 (1, N = 583) = 45.034, *p* < 0.001).

Independent samples *t*-tests comparing MetS cases and non-cases showed the former group had significantly higher BMI (kg/m2), exceeding the threshold for obesity (M = 33.41 versus 27.54), *t*(459.82) = -12.74, *p* < 0.001, greater waist/hip ratio (M = 0.98 versus 0.94), *t*(343.70) = -4.22, *p* < 0.001, and lower serum HDL-C (M = 1.18 versus 1.30), *t*(183.65) = 2.69, *p* < 0.01. There were no group differences in blood pressure, HbA1c, or lifestyle factors (all *p*’s > 0.01).

### Hypothesis 1: Does SRH predict metabolic abnormalities in T2DM patients?

Table 2 shows results of bootstrapped hierarchical multiple regression predicting metabolic abnormalities. SRH significantly predicted HDL-C (mmol/L) (Model 2) (β = -0.17, *p* = 0.015), increasing the explained variance, ∆*R*^2^ = 0.029, *F* [1, 176] = 6.035, *p* = 0.015. However, adjusting for metabolic factors (Model 3) negated this association, accounting for an additional 6.7% of the variance in HDL-C (∆*R*^2^ = 0.067, *F* (5, 171) = 2.976, *p* = 0.013).

**Table 2:** Final regression models predicting metabolic factors from self-rated health and metabolic covariates in the whole sample

	Outcome variables					
	Serum HDL cholesterol (mmol/L)	BMI (kg/m^2^)	Waist/hip ratio (cm)	Systolic blood pressure (mmHg)	Diastolic blood pressure (mmHg)	Glycated haemoglobin—HbA1c (mmol/mol)
Predictors (Model 3)	*B* 95%CI [LL, UL], *beta*	*B* 95%CI [LL, UL], *beta*	*B* 95%CI [LL, UL], *beta*	*B* 95%CI [LL, UL], *beta*	*B* 95%CI [LL, UL], *beta*	*B* 95%CI [LL, UL], *beta*
**Demographics, lifestyle factors**						
Age (three-year bands for 0–15, five-year bands for ages 16 +)	0.01 [-0.00, 0.03], 0.11	-0.36 [-0.70, -0.02], -0.17^a^	0.00 [0.00, 0.01], 0.20^b^	2.66 [1.77, 3.55], 0.45^c^	-1.67 [-2.22, -1.13], -0.41^c^	-0.52 [-1.69, 0.64], -0.07
Gender (male = 1, female = 0)	-0.08 [-0.20, 0.02], -0.13	-3.31 [-5.08, -1.54], -0.31^c^	0.09 [0.07, 0.11], 0.55^c^	3.84 [-1.33, 9.02], 0.11	-1.99 [-5.18, 1.19], -0.09	0.55 [-5.70, 6.80], 0.01
Socio-economic class (eight categories, coded 0 to 7: 0 = higher managerial/professional, 7 = never worked or unemployed)	-0.00 [-0.03, 0.03], -0.01	-0.00 [-0.54, 0.53], -0.00	0.00 [-0.00, 0.01], 0.05	0.73 [-0.78, 2.25], 0.06	-0.18 [-1.12, 0.75], -0.02	-0.21 [-2.04, 1.62], -0.01
Ethnicity (White = 1, non-white = 0)	-0.02 [-0.16, 0.12], -0.02	3.64 [1.43, 5.86], 0.24^c^	-0.00 [-0.03, 0.02], -0.03	-1.64 [-8.10, 4.82, -0.03	-3.54 [-7.48, 0.39], -0.11	4.76 [-2.97, 12.49], 0.09
Lifestyle factor: Smoking (number of cigarettes smoked per day)	-0.00 [-0.01, 0.00], -0.11	-0.02 [-0.11, 0.06], -0.03	0.00 [-0.00, 0.00], -0.03	0.09 [-0.16, 0.36], 0.04	-0.00 [-0.16, 0.16], -0.00	-0.01 [-0.33, 0.30], -0.00
Lifestyle factor: Alcohol consumption (frequency drunk in past 12 months)	-0.03 [-0.05, -0.01], -0.22^b^	0.24 [-0.10, 0.58], 0.10	-0.00 [-0.00, 0.00], -0.01	-0.23 [-1.21, 0.74], -0.03	0.01 [-0.59, 0.61], 0.00	-0.61 [-1.78, 0.55], -0.08
**Self-rated health** (very good/good = 1, fair/bad very bad = 0)	0.08 [-0.01, 0.17], 0.12	-0.39 [-1.92, 1.12], -0.03	-0.01 [-0.03, 0.00], -0.07	-2.22 [-6.53, 2.08], -0.06	0.81 [-1.84, 3.46], 0.03	-5.38 [-10.51, 0.25], -0.15^a^
**Anthropometric Markers**						
BMI (kg/m^2^)	-0.00 [-0.01, 0.00], -0.07	_	0.00 [0.00, 0.00], 0.22^c^	0.12 [-0.30, 0.55], 0.03	0.14 [-0.12, 0.40], 0.07	-0.02 [-0.54, 0.48], -0.00
Waist/hip ratio (cm)	-0.53 [-1.24, 0.17], -0.12	20.18 [9.13, 31.22], 0.31^c^	_	-1.81 [-34.28, 30.65], -0.00	6.39 [-13.54, 26.34], 0.04	18.05 [-20.84, 56.95], 0.08
**Biomarkers**						
Serum HDL cholesterol (mmol/L)	-	-1.24 [-3.65, 1.17], -0.07	-0.02 [-0.05, 0.00], -0.09	4.19 [-2.62, 11.01], 0.08	2.18 [-2.01, 6.38], 0.06	10.10 [-18.19, -2.02], -0.19^a^
Systolic blood pressure (mmHg)	0.00 [-0.00, 0.00], 0.10	0.01 [-0.03, 0.06], 0.04	0.00 [-0.00, 0.00], -0.00	_	0.31 [0.23, 0.39], 0.47^c^	-0.07 [-0.25, 0.10], -0.06
Diastolic blood pressure (mmHg)	0.00 [-0.00, 0.00], 0.08	0.04 [-0.04, 0.13], 0.09	0.00 [-0.00, 0.00], 0.04	0.82 [0.60, 1.03], 0.53^c^	_	0.43 [0.14, 0.72], 0.26^b^
Glycated haemoglobin—HbA1c (mmol/mol)	-0.00 [-0.00, -0.00], -0.17^a^	-0.00 [-0.04, 0.04], -0.00	0.00 [0.00, 0.00], 0.05	-0.05 [-0.17, 0.07], -0.05	0.11 [0.03, 0.18], 0.18^b^	_
*R*^*2*^* (adjusted R*^*2*^*)*	0.23 (0.18)	0.19 (0.14)	0.41 (0.37)	0.34 (0.29)	0.40 (0.36)	0.40 (0.16)
*F*	*F* (12, 171) = 4.35, *p* < 0.001	*F* (12, 171) = 3.51, *p* < 0.001	*F* (12, 171) = 9.94, *p* < 0.001	*F* (12, 171) = 7.47, *p* < 0.001	*F* (12, 171) = 9.86, *p* < 0.001	*F* (12, 171) = 2.84, *p* ≦ 0.001

*Note*. Model 1 (+ demographics, lifestyle factors), Model 2 (+ SRH), Model 3 (+ cardiometabolic covariates). Coefficients shown are from final step (Model 3)

^a^(*p* < 0.05), ^b^(*p* ≤ 0.01), ^c^(*p* ≤ 0.001)

SRH failed to predict systolic blood pressure (mmHg) (Model 2). Adding metabolic covariates (Model 3) significantly improved the model (∆*R*^2^ = 0.254, *F* (5, 171) = 13.269, *p* < 0.001), primarily due to diastolic covariance (β = 0.53, *p* < 0.001). Similarly, SRH failed to predict diastolic blood pressure (mmHg), whereas adding metabolic factors significantly improved model fit (∆*R*^2^ = 0.271, *F* (5, 171) = 15.660, *p* < 0.001), mainly due to systolic effects (β = 0.47, *p* < 0.001) and HbA1c (mmol/mol) (β = 0.18, *p* = 0.003).

The association between SRH and HbA1c (mmol/mol) was significant (β = -0.20, *p* = 0.008) prior to adjusting for metabolic covariates (Model 2) (∆*R*^2^ = 0.082, *F* (1, 176) = 7.241, *p* = 0.008). Adding metabolic variables (Model 3) significantly improved the model (∆*R*^2^ = 0.084, *F* (5, 171) = 3.454, *p* = 0.005), negating the SRH − HbA1c relationship (*p* = 0.04). Finally, SRH failed to predict anthropometric criteria (BMI, (kg/m^2^), waist/hip ratio (cm)) (Model 2). Including metabolic factors explained additional variance for both BMI (∆*R*^2^ = 0.090, *F* (5, 171) = 3.835, *p* = 0.003) and waist/hip ratio (∆*R*^2^ = 0.069 *F* (5, 171) = 4.027, *p* = 0.002).

### Hypothesis 2: Does SRH predict metabolic abnormalities in T2DM patients by MetS status?

Table 3 shows the results for T2DM patients who met MetS diagnostic criteria. Crucially, SRH failed to predict any metabolic variable (Model 2) prior to adjusting for metabolic covariates (Model 3) (all *p*’s > 0.01).

**Table 3:** Final regression models predicting metabolic factors from self-rated health and metabolic covariates in T2DM patients with MetS

	Outcome variables					
	Serum HDL cholesterol (mmol/L)	BMI (kg/m^2^)	Waist/hip ratio (cm)	Systolic blood pressure (mmHg)	Diastolic blood pressure (mmHg)	Glycated haemoglobin—HbA1c (mmol/mol)
Predictors (Model 3)	*B* 95%CI [LL, UL], *beta*	*B* 95%CI [LL, UL], *beta*	*B* 95%CI [LL, UL], *beta*	*B* 95%CI [LL, UL], *beta*	*B* 95%CI [LL, UL], *beta*	*B* 95%CI [LL, UL], *beta*
**Demographics, Lifestyle factors**						
Age (three-year bands for 0–15, five-year bands for ages 16 +)	0.00 [-0.04, 0.04], 0.00	-0.75 [-1.21, -0.30], -0.44^c^	0.00 [-0.00, 0.01], 0.17	2.01 [0.17, 3.84], 0.29^a^	-1.78 [-2.77, -0.80], -0.41^c^	-1.59 [-3.88, 0.68], -0.20
Gender (male = 1, female = 0)	-0.09 [-0.34, 0.14], -0.14	-3.59 [-6.26, 0.92], -0.42^b^	0.12 [0.09, 0.16], 0.74c	3.53 [-7.38, 14.44], 0.10	0.66 [-5.58, 6.91], 0.03	-5.18 [-18.48, 8.11], -0.13
Socio-economic class (eight categories, coded 0 to 7: 0 = higher managerial/professional, 7 = never worked or unemployed)	0.01 [-0.05, 0.07], 0.03	-0.21 [-0.93, 0.50], -0.06	0.00 [-0.00, 0.02], 0.12	-1.32 [-4.10, 1.45], -0.10	-0.21 [-1.81, 1.38], -0.02	-1.16 [-4.57, 2.23], -0.07
Lifestyle factor: Smoking (number of cigarettes smoked per day)	-0.00 [-0.01, 0.00], -0.08	-0.05 [-0.16, 0.06], -0.10	0.00 [-0.00, 0.00], -0.02	-0.14 [-0.58, 0.30], -0.06	0.02 [-0.23, 0.27], 0.01	-0.29 [-0.83, 0.24], -0.12
Lifestyle factor: Alcohol consumption (frequency drunk in past 12 months)	-0.03 [-0.07, 0.00], 0.23	0.23 [-0.20, 0.67], 0.13	-0.00 [-0.00, 0.00, -0.03	0.78 [-0.93, 2.50], 0.10	0.00 [-0.98, 0.98], 0.00	-1.12 [-3.22, 0.96], -0.13
**Self-rated health** (very good/good = 1, fair/bad very bad = 0)	0.09 [-0.07, 0.25], 0.13	-0.19 [-2.10, 1.71], -0.02	-0.01 [-0.04, 0.01], -0.07	1.51 [-5.88, 8.91], 0.04	-0.33 [-4.56, 3.89], -0.01	-6.26 [-15.16, 2.63], -0.16
**Anthropometric Markers**						
BMI (kg/m^2^)	-0.00 [-0.02, 0.01], -0.05	_	0.00 [0.00, 0.00], 0.19^a^	0.16 [-0.81, 1.13], 0.04	0.25 [-0.30, 0.80], 0.10	-0.91 [-2.08, 0.25], -0.19
Waist/hip ratio (cm)	-0.49 [-1.83, 0.84], -0.12	15.41 [0.22, 30.59], 0.31^a^	_	16.21 [-44.50, 76.93], 0.08	-16.83 [-51.30, 17.63], -0.13	28.95 [-44.93, 102.84], 0.12
**Biomarkers**						
Serum HDL cholesterol (mmol/L)	_	-0.61 [-3.54, 2.31], -0.05	-0.01 [-0.06, 0.03], -0.07	6.82 [-4.43, 18.08], 0.14	1.11 [-5.37, 7.61], 0.03	-12.94 [-26.45, 0.56], -0.23
Systolic blood pressure (mmHg)	0.00 [-0.00, 0.00], 0.16	0.01 [-0.05, 0.07], 0.04	0.00 [-0.00, 0.00], 0.05	_	0.29 [0.16, 0.41], 0.45^c^	-0.16 [-0.47, 0.13], -0.14
Diastolic blood pressure (mmHg)	0.00 [-0.00, 0.01], 0.05	0.05 [-0.06, 0.16], 0.13	-0.00 [-0.00, 0.00], -0.11	0.88 [0.50, 1.26], 0.56^c^	_	0.43 [-0.09, 0.96], 0.23
Glycated haemoglobin—HbA1c (mmol/mol)	-0.00 [-0.00, 0.00], -0.23	-0.04 [-0.09, 0.01], -0.18	0.00 [-0.00, 0.00], 0.07	-0.11 [-0.31, 0.09], -0.12	0.09 [-0.02, 0.21], 0.17	_
*R*^*2*^* (adjusted R*^*2*^*)*	0.24 (0.11)	0.29 (0.17)	0.56 (0.49)	0.34 (0.23)	0.46 (0.37)	0.26 (0.13)
*F*	*F* (11, 63) = 1.85, *p* > 0.05	*F* (11, 63) = 2.44, *p* < 0.05	*F* (11, 63) = 7.59, *p* < 0.001	*F* (11, 63) = 3.03, *p* < 0.01	*F* (11, 63) = 4.99, *p* < 0.001	*F* (11, 63) = 2.01, *p* < 0.05

*Note*. Model 1 (+ demographics, lifestyle factors), Model 2 (+ SRH), Model 3 (+ cardiometabolic covariates). Coefficients shown are from final step (Model 3). Ethnicity was excluded due to low frequencies for non-whites [check this]

^a^(*p* < 0.05), ^b^(*p* ≤ 0.01), ^c^(*p* ≤ 0.001)

BMI was predicted by both age (β = -0.44, *p* = 0.001) and gender (β = -0.42, *p* = 0.009). Gender also predicted waist/hip ratio (*p* < 0.001), while age predicted diastolic blood pressure (*p* = 0.001). Adding metabolic predictors (Model 3) significantly improved the predicted variance for systolic blood pressure (∆*R*^2^ = 0.286, *F* (5, 63) = 5.517, *p* < 0.001) and diastolic blood pressure (∆*R*^2^ = 0.229, *F* (5, 63) = 5.395, *p* < 0.001).

Table 4 shows coefficients for patients who did *not* meet MetS criteria (i.e., T2DM-only patients). Again, SRH failed to predict any metabolic factor (Model 2), prior to accounting for metabolic covariates (all *p*’s > 0.01). Adjusting for metabolic variables (Model 3) explained significant additional variance for both systolic (∆*R*^2^ = 0.211, *F* (5, 96) = 7.069, *p* < 0.001) and diastolic (∆*R*^2^ = 0.286, *F* (5, 96) = 9.236, *p* < 0.001) blood pressure.

**Table 4:** Final regression models predicting metabolic factors from self-rated health and metabolic covariates in T2DM patients without MetS

	Outcome variables					
	Serum HDL cholesterol (mmol/L)	BMI (kg/m^2^)	Waist/hip ratio (cm)	Systolic blood pressure (mmHg)	Diastolic blood pressure (mmHg)	Glycated haemoglobin—HbA1c (mmol/mol)
Predictors (Model 3)	*B* 95%CI [LL, UL], *beta*	*B* 95%CI [LL, UL], *beta*	*B* 95%CI [LL, UL], *beta*	*B* 95%CI [LL, UL], *beta*	*B* 95%CI [LL, UL], *beta*	*B* 95%CI [LL, UL], *beta*
**Demographics, Lifestyle factors**						
Age (three-year bands for 0–15, five-year bands for ages 16 +)	0.02 [-0.00, 0.05], 0.19	0.02 [-0.36, 0.40], 0.01	0.00 [0.00, 0.01], 0.30^b^	2.72 [1.60, 3.83], 0.45^c^	-1.51 [-2.28, -0.73], -0.38^c^	-0.62 [-2.09, 0.83], -0.10
Gender (male = 1, female = 0)	-0.09 [-0.23, 0.04], -0.14	-2.34 [-4.15, -0.53], -0.28^a^	0.06 [0.03, 0.09], 0.43^c^	1.97 [-4.04, 7.99], 0.06	-2.92 [-6.90, 1.06], -0.13	2.74 [-4.35, 9.84], 0.08
Socio-economic class (eight categories, coded 0 to 7: 0 = higher managerial/professional, 7 = never worked or unemployed)	-0.01 [-0.05, 0.03], -0.05	0.39 [-0.19, 0.98], 0.13	0.00 [-0.00, 0.01], 0.04	1.92 [0.03, 3.81], 0.16^a^	-0.38 [-1.66, 0.90], -0.04	0.28 [-1.98, 2.56], 0.02
Ethnicity (White = 1, non-white = 0)	0.00 [-0.16, 0.17], 0.00	1.77 [-0.51, 4.06], 0.17	-0.00 [-0.04, 0.03], -0.03	1.26 [-6.21, 8.75], 0.03	-5.45 [-10.32, -0.57], -0.20^a^	7.67 [-1.02, 16.37], 0.19
Lifestyle factor: Smoking (number of cigarettes smoked per day)	-0.00 [-0.01, 0.00], -0.15	-0.00 [-0.11, 0.10], -0.01	0.00 [-0.00, 0.00], -0.04	0.17 [-0.17, 0.51], 0.08	-0.04 [-0.27, 0.19], -0.03	0.25 [-0.15, 0.66], 0.12
Lifestyle factor: Alcohol consumption (frequency drunk in past 12 months)	-0.03 [-0.05, -0.00], -0.22^a^	0.13 [-0.24, 0.51], 0.07	0.00 [-0.00, 0.00], 0.00	-0.51 [-1.73, 0.71], -0.07	0.01 [-0.80, 0.83], 0.00	0.44 [-0.99, 1.89], 0.06
**Self-rated health** (very good/good = 1, fair/bad very bad = 0)	0.06 [-0.06, 0.18], 0.09	-0.27 [-1.98, 1.43], -0.03	-0.01 [-0.03, 0.01], -0.06	-4.52 [-9.95, 0.91], -0.13	2.16 [-1.49, 5.81], 0.10	-3.35 [-9.83, 3.11], -0.10
**Anthropometric Markers**						
BMI (kg/m^2^)	-0.00 [-0.01, 0.01], -0.04	_	0.00 [-0.00, 0.00], 0.13	-0.17 [-0.82, 0.48], -0.04	0.02 [-0.41, 0.46], 0.00	0.15 [-0.61, 0.93], 0.04
Waist/hip ratio (cm)	-0.57 [-1.50, 0.36], -0.13	9.33 [-3.12, 21.79], 0.17	_	-9.32 [-49.94, 31.29], -0.04	19.17 [-7.69, 46.04], 0.13	15.81 [-32.07, 63.70], 0.07
**Biomarkers**						
Serum HDL cholesterol (mmol/L)	_	-0.69 [-3.40, 2.01], -0.05	-0.02 [-0.07, 0.01], -0.11	1.50 [-7.23, 10.23], 0.03	2.97 [-2.82, 8.78], 0.09	-5.57 [-15.82, 4.67], -0.11
Systolic blood pressure (mmHg)	0.00 [-0.00, 0.00], 0.04	-0.01 [-0.07, 0.04], -0.06	0.00 [-0.00, 0.00], -0.05	_	0.32 [0.21, 0.44], 0.50^c^	0.01 [-0.22, 0.25], 0.01
Diastolic blood pressure (mmHg)	0.00 [-0.00, 0.01], 0.11	0.00 [-0.08, 0.09], 0.01	0.00 [0.00, 0.00], 0.15	0.73 [0.47, 1.00], 0.48^c^	_	0.40 [0.05, 0.75], 0.27^a^
Glycated haemoglobin—HbA1c (mmol/mol)	-0.00 [-0.00, 0.00], -0.10	0.01 [-0.04, 0.06], 0.04	0.00 [-0.00, 0.00], 0.06	0.01 [-0.15, 0.18], 0.01	0.13 [0.01, 0.24], 0.19^a^	_
*R*^*2*^* (adjusted R*^*2*^*)*	0.21 (0.12)	0.12 (0.01)	0.33 (0.25)	0.42 (0.35)	0.40 (0.33)	0.17 (0.06)
*F*	*F* (12, 96) = 2.24, *p* < 0.05	*F* (12, 96) = 1.14, *p* > 0.05	*F* (12, 96) = 3.99, *p* < 0.001	*F* (12, 96) = 5.93, *p* < 0.001	*F* (12, 96) = 5.47, *p* < 0.001	*F* (12, 96) = 1.66, *p* > 0.05

*Note*. Model 1 (+ demographics, lifestyle factors), Model 2 (+ SRH), Model 3 (+ cardiometabolic covariates). Coefficients shown are from final step (Model 3)

^a^(*p* < 0.05), ^b^(*p* ≤ 0.01), ^c^(*p* ≤ 0.001)

### Exploratory analysis by age and gender

Research suggests gender differences in cardiometabolic risk [54, 55]. Given that gender was associated with metabolic covariates (see Table 2), we decided to rerun regression analysis separately for males and females. Significant patterns emerged for HDL-C and HbA1c. These results are shown in Table 5.

**Table 5:** Final regression models predicting HDL-C and HbA1c from self-rated health and metabolic covariates in males and females

	Outcome variables			
	Serum HDL cholesterol (mmol/L)		Glycated haemoglobin—HbA1c (mmol/mol)	
	Female	Male	Female	Male
Predictors (Model 3)	*B* 95%CI [LL, UL], *beta*	*B* 95%CI [LL, UL], *beta*	*B* 95%CI [LL, UL], *beta*	*B* 95%CI [LL, UL], *beta*
**Demographics, lifestyle**				
Age (three-year bands for 0–15, five-year bands for ages 16 +)	0.03 [0.00, 0.06], 0.29^a^	-0.01 [-0.05, 0.01], -0.13	0.07 [-1.46, 1.62], 0.01	-2.22 [-4.06, -0.37], -0.29^b^
Socio-economic class (eight categories, coded 0 to 7: 0 = higher managerial/professional, 7 = never worked or unemployed)	0.00 [-0.04, 0.05], 0.01	-0.02 [-0.06, 0.02], -0.08	-0.72 [-3.35, 1.90], -0.05	-0.47 [-3.04, 2.08], -0.03
Ethnicity (White = 1, non-white = 0)	-0.27 [-0.52, -0.03], -0.27^a^	0.11 [-0.07, 0.29], 0.12	2.87 [-10.02, 15.76], 0.05	3.63 [-6.70, 13.98], 0.07
Lifestyle factor: Smoking (number of cigarettes smoked per day)	-0.00 [-0.01, 0.00], -0.10	-0.00 [-0.01, 0.00], -0.12	0.07 [-0.36, 0.50], 0.03	-0.01 [-0.47, 0.44], -0.00
Lifestyle factor: Alcohol consumption (frequency drunk in past 12 months)	-0.03 [-0.06, -0.00], -0.23^a^	-0.03 [-0.05, 0.00], -0.20^a^	0.54 [-1.17, 2.27], 0.07	-1.89 [-3.51, -0.26], -0.24^a^
**Self-rated health** (very good/good = 1, fair/bad very bad = 0)	0.02 [-0.12, 0.17], 0.04	0.16 [0.03, 0.29], 0.25^b^	-9.30 [-16.68, -1.93], -0.27^b^	0.68 [-6.59, 7.97], 0.01
**Cardiometabolic factors**				
BMI (kg/m^2^)	0.00 [-0.01, 0.01], 0.02	-0.01 [-0.03, 0.00], -0.22	0.43 [-0.28, 1.15], 0.13	-0.93 [-1.81, -0.05], -0.26^a^
Waist/hip ratio (cm)	-1.12 [-2.14, -0.09], -0.23^a^	0.19 [-0.99, 1.39], 0.04	17.25 [-36.56, 71.07], 0.07	45.01 [-20.80, 110.83], 0.16
Serum HDL cholesterol (mmol/L)	_	_	-9.61 [-21.37, 2.13], -0.18	-14.09 [-25.52, -2.66], -0.25^b^
Systolic blood pressure (mmHg)	0.00 [-0.00, 0.00], 0.17	0.00 [-0.00, 0.00], 0.11	-0.01 [-0.26, 0.24], -0.01	-0.06 [-0.32, 0.20], -0.05
Diastolic blood pressure (mmHg)	0.00 [-0.00, 0.01], 0.06	0.00 [-0.00, 0.01], 0.05	0.45 [0.08, 0.83], 0.31^b^	0.28 [-0.18, 0.75], 0.15
Glycated haemoglobin—HbA1c (mmol/mol)	-0.00 [-0.00, 0.00], -0.18	-0.00 [-0.00, -0.00], -0.24^b^	_	_
*R*^*2*^* (adjusted R*^*2*^*)*	0.32 (0.21)	0.25 (0.16)	0.31 (0.21)	0.21 (0.11)
*F*	*F* (11, 72) = 3.10, *p* < 0.01	*F* (11, 88) = 2.73, *p* < 0.01	*F* (11, 72) = 3.04, *p* < 0.01	*F* (11, 88) = 2.21, *p* < 0.05

*Note*. Model 1 (+ demographics, lifestyle factors), Model 2 (+ SRH), Model 3 (+ cardiometabolic covariates). Coefficients shown are from final step (Model 3)

^a^(*p* < 0.05), ^b^(*p* ≤ 0.01), ^c^(*p* ≤ 0.001)

SRH significantly predicted HDL-C (mmol/L) in male patients (Model 2) (β = 0.25, *p* = 0.01), accounting for a significant 6.1% increase in the explained variance, after accounting for demographic and lifestyle factors, ∆*R*^2^ = 0.061, *F* (1, 93) = 6.712, *p* = 0.011. Adjusting for metabolic factors (Model 3) did not negate the association between SRH and HDL-C (β = 0.25, *p* = 0.01) in males and failed to improve the model (∆*R*^2^ = 0.095, *F* (5, 88) = 2.253, *p* = 0.056). SRH also predicted HbA1c (mmol/mol) in female patients (Model 2) (β = -0.31, *p* = 0.007), explaining 8.4% variance (∆*R*^2^ = 0.084, *F* (1, 77) = 7.696, *p* = 0.007). Adjusting for metabolic abnormalities (Model 3) significantly improved the model, predicting another 15% of the variance (∆*R*^2^ = 0.156, *F* (5, 72) = 3.287, *p* = 0.01), but did not nullify the SRH − HbA1c association (β = -0.27, *p* = 0.01). SRH failed to predict the other metabolic variables, irrespective of metabolic adjustment (all *p*’s > 0.01).

Regardless, the associations of SRH with HDL-C (in men) and HbA1c (in women) were not significant based on the Bonferroni adjusted alpha level (both *p*’s > 0.004).

Given that age is strongly implicated in metabolic health [56], and was also significantly associated with various metabolic covariates, notably systolic/diastolic blood pressure (see Table 2), we repeated the analysis, to see whether SRH significantly predicts metabolic variables across older (≥ age 65) and younger (< age 65) respondents, based on a median split. SRH was not reliably associated with any metabolic outcome, irrespective of age group (all *p*'s > 0.004).

### Sensitivity analysis

We reanalysed the data with expectation maximisation applied to missing values, to compare the effects of different methods for resolving incomplete data (list wise deletion versus EM). As observed in previous analysis, SRH failed to predict HDL-C (mmol/L), waist/hip ratio (cm), and systolic/diastolic blood pressure (mmHg) after adjusting for metabolic covariates (all *p*’s > 0.01). However, contrary to expectations, SRH significantly predicted BMI (kg/m2) after metabolic adjustment (Model 3) (β = -0.12, *p* = 0.002). Furthermore, the previously significant SRH − HbA1c association was no longer reliable (β = -0.06, *p* = 0.10). Collapsing the data by MetS status (cases versus non-cases) did not change the results: SRH failed to predict any metabolic variable after adjusting for metabolic covariates (Model 3) (all *p*’s > 0.004). Overall, sensitivity analysis indicated most findings were unaffected by the management of missing data using expectation maximisation algorithms.

### Structural equation modelling

We used SEM to explore direct and indirect associations between SRH and metabolic abnormities. We were curious to see whether relations between SRH and metabolic factors are indirect, mediated by lifestyle factors (e.g., SRH negates health-protective behaviours, which in turn precipitate metabolic dysfunction) [8]. Model fit was based on standard criteria: chi-square χ2 (CMIN) (*p* > 0.05), χ2 (CMIN)/df < 5.00, root mean square error of approximation (RMSEA) < 0.07, comparative fit index (CFI) ≥ 0.95, Tucker and Lewis Index (TLI) ≥ 0.95, and normed fit index (NFI) ≥ 0.95 [57]. Metabolic factors were allowed to affect SRH, that in turn was allowed to predict lifestyle factors, which then affected metabolic variables (representing a vicious cycle in which lifestyle was a mediating factor). SEM analysis using IBM SPSS AMOS™ (version 26), with specification search, generated 192 candidate models, none of which provided a satisfactory fit. The ‘best’ model (BIC (Bayesian Information Criterion) = 0, χ2 (CMIN)/df < 5.00) suggested a cyclical relationship between HDL-C, SRH, and alcohol intake. However, this model did not satisfy most other fit criteria: CMIN (*p* < 0.05), RMSEA (> 0.07), CFI (< 0.95), and TLI (< 0.95)) and was therefore discarded.

## Discussion

There is currently a lack of research on psychosocial tools that primary care physicians can use for detecting metabolic abnormalities in people diagnosed with T2DM. Overall, we found little evidence SRH reliably predicts metabolic dysfunction in T2DM patients, after accounting for metabolic covariates. This finding contradicts previous population-based study suggesting SRH independently predicts metabolic variables, irrespective of health status [10]. Although that investigation controlled for physical illness (e.g., number of diseases), there was no adjustment metabolic covariates. We argued this was problematic given metabolic comorbidity [29–31], which may partly explain reported associations between SRH and biomarkers. Our findings suggest the contribution of SRH to HDL-C and HbA1c when stratified by gender is notable but negligible in the context of clinical biomarkers. SRH may simply be a psychological manifestation of metabolic comorbidity [30, 31]. For example, given widespread awareness of HbA1c and its relevance in glycaemic control [58], a poor HbA1c test result (or symptoms suggesting hyperglycaemia) is likely to be viewed as a sign of poor health by most T2DM patients [59]. Poor SRH may also reflect feedback from other cardiometabolic tests highlighting metabolic dysfunction [60].

Future research needs to explore the role of gender in the relationship between SRH and metabolic health. Evidence suggests women are less likely to achieve HbA1c targets, which may their affect health judgements. Women with diabetes are also more prone to blood sugar changes overnight (nocturnal hypoglycaemia) [61], which perhaps may contribute to health evaluations. Thus, there is a need to better understand women's greater sensitivity to HbA1c, and whether SRH might be a useful indicator of poor glycaemic control in certain female T2DM patients, irrespective of related metabolic abnormalities. This diagnostic utility becomes especially relevant if HbA1c is used to define MetS [41]. It is also necessary to determine whether men and women use similar frames of reference when making judgements about their health [62]. For example, evidence suggests cholesterol management is worse in women [63], including those with T2DM, and women with T2DM less frequently achieve cholesterol targets compared with men [64]. This suggests male and female T2DM patients may have very different perceptions of health based on varied cardiometabolic profiles [65].

Despite a slight tendency for MetS cases to be older, age played no role in the association between SRH and metabolic health. This is a curious finding given that age and metabolic health are inextricably connected [56]. Interestingly, previous studies with young people have found SRH reliably predicts both mortality [14] and morbidity [15], despite their better health status. However, it should be noted that some of this research examined disease conditions characterised by overt symptoms or pain, such as infections, allergy and injuries [15], which people are likely to perceive as indications of poor health. By contrast, the *asymptomatic* nature of some cardiometabolic dysfunctions, such as hypertension [22] and obesity [23], means people's SRH may not adequately capture underlying metabolic abnormalities, regardless of their age.

Interestingly, the relationship between SRH and metabolic factors was unaffected by MetS status. The concept of MetS as a distinct illness may have limited *psychological* relevance in T2DM. There is considerable ambiguity even amongst health professionals regarding what defines MetS, and different criteria have been proposed [2, 5]. Awareness of MetS is low, amongst both health care providers [66] and people at high risk [67]. Thus, diagnostic metabolic dysfunctions may not be experienced by T2DM patients as a sign of poor health. Furthermore, it is notable the regression models (*R*^*2*^ values) were particularly weak in predicting outcomes amongst patients who did *not* meet MetS criteria. Demographic factors, notably age and gender, seemed particularly relevant in this group. Unfortunately, the biological mechanisms underpinning gender differences, aging, and longevity, are complicated and poorly understood [68, 69], and more research is needed to better understand the interrelationships between demographic factors, SRH, and metabolic dysregulation in T2DM patients.

### Implications for primary care

Although management of type 2 diabetes (T2DM) typically occurs in primary care settings [1], and physicians are tasked with using a ‘whole person’ approach [7], there has been a paucity of evidence-based psychosocial diagnostic tools for detecting metabolic dysfunction in T2DM patients. Our data suggests T2DM patients incorporate HDL-C and HbA1c anomalies into their subjective health assessments. While this suggests SRH can be used to screen for HDL-C deficiency in male patients, and elevated HbA1c concentrations in female patients, before they have developed overt clinical metabolic dysfunction [8], the added diagnostic value over clinical data is marginal at best. This raises an important question: should T2DM patients be asked to rate their own health during routine medical assessments or consultations with their primary care physician, pending further research? As this was a single-cohort study with sex-stratified analyses, more research is needed to further explore the gender-specific themes. For example, it remains unclear from the current data whether female patients with poor SRH need to be prioritised for further blood tests, to measure HbA1c levels, or male patients with bleak SRH should be recommended for HDL-C testing. Future studies should focus on the association between SRH and lipid profiles [10]. Unlike high blood sugar, which generates overt symptoms such as increased thirst, fatigue, or frequent urination, patients with high cholesterol don’t typically show any symptoms, and hence can be sent for further clinical assessment if they disclose poor SRH [6].

### Limitations

This study did not assess triglycerides (> 1.7 mmol/L (150 mg/dl), which is an important diagnostic criterion for MetS [2]. Also, the analysis of HbA1c in place of fasting glucose is debatable [5], albeit this reflects new MetS diagnostic criteria proposed by the IDF [41]. The assumption insulin resistance defines T2DM is problematic. Although poor insulin sensitivity is characteristic of T2DM, it may not apply to nonobese patients (circa 10–15% of T2DM patients) [43]. Overall, it remains unclear how direct measures of insulin resistance, fasting glucose, and triglycerides would have impacted the current findings. Given the paucity of independent associations between SRH and metabolic factors in the current data, it is unlikely adjusting for these additional biomarkers will dramatically alter the results. Nevertheless, complex mediator effects are possible, and future research needs to further explore viable indirect pathways, using SEM. Sensitivity analysis showed that most findings were unaffected by the type of algorithm used to manage missing data. One notable exception was a previously non-significant association between SRH and BMI (kg/m^2^), which became significant after applying the expectation maximisation method. While this algorithm may generate biased estimates and models [52], it is nevertheless essential that future research authenticate the current findings by comparing different methods of handling incomplete data. Another issue is that the Bonferroni adjustment may have increased the risk of a false negatives [48]. Finally, as this was a single-cohort study the findings require replication in another cohort using the same research design.

### Conclusions

While primary care professionals have a growing plethora of clinical options for detecting metabolic abnormalities in T2DM, there has been limited research on useful psychological tools for detecting metabolic dysfunction in this clinical population, despite the emphasis on a holistic approach in primary care. This is the first study to assess the link between SRH and metabolic dysfunction in T2DM patients, while accounting for metabolic comorbidity. Overall, our findings suggest that while SRH may help primary care physicians identify T2DM patients with HDL-C and HbA1c abnormalities, the added diagnostic utility over clinical biomarkers is negligible.

## Data Availability

The Health Survey for England (HSE) is managed by the National Centre for Social Research (NatCen) and the Department of Epidemiology and Public Health at University College London. HSE data cannot be shared publicly for legal and ethical reasons, third party rights, and institutional or national regulations or laws. The UK Data Service provides restricted access to HSE data, to protect confidential or proprietary information. Individuals and organisations seeking access need to be registered with the UK Data Service, albeit access is limited to applicants from UK HE/FE institutions, central and local government, NHS, research companies and charities for not-for-profit education and research purposes. Users not in the above categories can submit access requests to surveys.queries@nhs.net and will be subject to approval. For more information, please contact the UK Data Service website. https://rb.gy/vhi5uf.

## References

[1] Seidu S, Cos X, Brunton S, Harris SB, Jansson SPO, Mata-Cases M, et al. 2022 update to the position statement by Primary Care Diabetes Europe: a disease state approach to the pharmacological management of type 2 diabetes in primary care. Prim Care Diabetes. 2022;16(2):223–44. 35183458 10.1016/j.pcd.2022.02.002

[2] Saklayen MG. The global epidemic of the metabolic syndrome. Curr Hypertens Rep. 2018;20(2):12. 29480368 10.1007/s11906-018-0812-z PMC5866840

[3] Alberti KG, Eckel RH, Grundy SM, Zimmet PZ, Cleeman JI, Donato KA, et al. Harmonizing the metabolic syndrome: a joint interim statement of the International Diabetes Federation Task Force on Epidemiology and Prevention; National Heart, Lung, and Blood Institute; American Heart Association; World Heart Federation; International Atherosclerosis Society; and International Association for the Study of Obesity. Circulation. 2009;120(16):1640–5. 19805654 10.1161/CIRCULATIONAHA.109.192644

[4] Regufe VMG, Pinto C, Perez P. Metabolic syndrome in type 2 diabetic patients: a review of current evidence. Porto Biomed J. 2020;5(6): e101. 33299950 10.1097/j.pbj.0000000000000101 PMC7721212

[5] Rochlani Y, Pothineni NV, Kovelamudi S, Mehta JL. Metabolic syndrome: pathophysiology, management, and modulation by natural compounds. Ther Adv Cardiovasc Dis. 2017;11(8):215–25. 28639538 10.1177/1753944717711379 PMC5933580

[6] Swarup S, Goyal A, Grigorova Y, Zeltser R. Metabolic Syndrome. Treasure Island: StatPearls Publishing LLC; 2022. 29083742

[7] Juanamasta IG, Aungsuroch Y, Gunawan J, Suniyadewi NW, NopitaWati NM. Holistic care management of diabetes mellitus: an integrative review. Int J Prev Med. 2021;12:69. 34447511 10.4103/ijpvm.IJPVM_402_20 PMC8356953

[8] Idler EL, Benyamini Y. Self-rated health and mortality: a review of twenty-seven community studies. J Health Soc Behav. 1997;38(1):21–37. 9097506

[9] Schnittker J, Bacak V. The increasing predictive validity of self-rated health. PLoS ONE. 2014;9(1):e84933. 24465452 10.1371/journal.pone.0084933 PMC3899056

[10] Kananen L, Enroth L, Raitanen J, Jylhava J, Burkle A, Moreno-Villanueva M, et al. Self-rated health in individuals with and without disease is associated with multiple biomarkers representing multiple biological domains. Sci Rep. 2021;11(1):6139. 33731775 10.1038/s41598-021-85668-7 PMC7969614

[11] Bombak AE. Self-rated health and public health: a critical perspective. Front Public Health. 2013;1:15. 24350184 10.3389/fpubh.2013.00015 PMC3855002

[12] Latham K, Peek CW. Self-rated health and morbidity onset among late midlife U.S. adults. J Gerontol B Psychol Sci Soc Sci. 2013;68(1):107–16. 23197340 10.1093/geronb/gbs104 PMC3605944

[13] Wu S, Wang R, Zhao Y, Ma X, Wu M, Yan X, et al. The relationship between self-rated health and objective health status: a population-based study. BMC Public Health. 2013;13:320. 23570559 10.1186/1471-2458-13-320 PMC3637052

[14] Vie TL, Hufthammer KO, Meland E, Breidablik HJ. Self-rated health (SRH) in young people and causes of death and mortality in young adulthood. A prospective registry-based Norwegian HUNT-study. SSM Popul Health. 2019;7:100364. 30723772 10.1016/j.ssmph.2019.100364 PMC6351583

[15] Hetlevik O, Meland E, Hufthammer KO, Breidablik HJ, Jahanlu D, Vie TL. Self-rated health in adolescence as a predictor of “multi-illness” in early adulthood: A prospective registry-based Norwegian HUNT study. SSM Popul Health. 2020;11:100604. 32509958 10.1016/j.ssmph.2020.100604 PMC7265049

[16] Liu Y, Ozodiegwu ID, Nickel JC, Wang K, Iwasaki LR. Self-reported health and behavioral factors are associated with metabolic syndrome in Americans aged 40 and over. Prev Med Rep. 2017;7:193–7. 28725542 10.1016/j.pmedr.2017.06.010 PMC5503882

[17] Lee BG, Lee JY, Kim SA, Son DM, Ham OK. Factors associated with self-rated health in metabolic syndrome and relationship between sleep duration and metabolic syndrome risk factors. J Korean Acad Nurs. 2015;45(3):420–8. 26159143 10.4040/jkan.2015.45.3.420

[18] Botoseneanu A, Ambrosius WT, Beavers DP, de Rekeneire N, Anton S, Church T, et al. Prevalence of metabolic syndrome and its association with physical capacity, disability, and self-rated health in Lifestyle Interventions and Independence for Elders Study participants. J Am Geriatr Soc. 2015;63(2):222–32. 25645664 10.1111/jgs.13205 PMC4333053

[19] Okosun IS, Airhihenbuwa C, Henry TL. Allostatic load, metabolic syndrome and self-rated health in overweight/obese Non-Hispanic White, non-Hispanic Black and Mexican American adults. Diabetes Metab Syndr. 2021;15(4):102154. 34186341 10.1016/j.dsx.2021.05.027

[20] Kim MH, Chang Y, Jung HS, Shin H, Ryu S. Impact of self-rated health on progression to a metabolically unhealthy phenotype in metabolically healthy obese and non-obese individuals. J Clin Med. 2019;8(1):34. 30609650 10.3390/jcm8010034 PMC6352103

[21] Kim MJ, Kim IW. Self-rated health may be a predictor for metabolic syndrome and high hs-CRP prevalences in healthy adults in South Korea: Based on the 2015 Korea National Health and Nutrition Examination Survey. Nutr Res. 2022;102:71–83. 35436679 10.1016/j.nutres.2022.03.003

[22] Gauer R. Severe Asymptomatic Hypertension: Evaluation and Treatment. Am Fam Physician. 2017;95(8):492–500. 28409616

[23] Robinson E. Overweight but unseen: a review of the underestimation of weight status and a visual normalization theory. Obes Rev. 2017;18(10):1200–9. 28730613 10.1111/obr.12570 PMC5601193

[24] Shirom A, Toker S, Melamed S, Shapira I. The relationships between self-rated health and serum lipids across time. Int J Behav Med. 2012;19(1):73–81. 21302015 10.1007/s12529-011-9144-y

[25] Tomten SE, Hostmark AT. Self-rated health showed a consistent association with serum HDL-cholesterol in the cross-sectional Oslo Health Study. Int J Med Sci. 2007;4(5):278–87. 18071582 10.7150/ijms.4.278 PMC2096714

[26] Uchino BN. Self-rated health and age-related differences in ambulatory blood pressure: the mediating role of behavioral and affective factors. Psychosom Med. 2020;82(4):402–8. 32150013 10.1097/PSY.0000000000000795 PMC7196491

[27] Shin HY, Shin MH, Rhee JA. Gender differences in the association between self-rated health and hypertension in a Korean adult population. BMC Public Health. 2012;12:135. 22340138 10.1186/1471-2458-12-135 PMC3306731

[28] Shankar A, Wang JJ, Rochtchina E, Mitchell P. Association between self-rated health and incident severe hypertension among men: a population-based cohort study. Singapore Med J. 2008;49(11):860–7. 19037550

[29] Kim YJ, Hwang HR. Clustering effects of metabolic factors and the risk of metabolic syndrome. J Obes Metab Syndr. 2018;27(3):166–74. 31089559 10.7570/jomes.2018.27.3.166 PMC6504198

[30] Parapid B, Ostojic MC, Lalic NM, Micic D, Damjanovic S, Bubanja D, et al. Risk factors clustering within the metabolic syndrome: a pattern or by chance? Hellenic J Cardiol. 2014;55(2):92–100. 24681786

[31] Melka MG, Abrahamowicz M, Leonard GT, Perron M, Richer L, Veillette S, et al. Clustering of the metabolic syndrome components in adolescence: role of visceral fat. PLoS ONE. 2013;8(12): e82368. 24376531 10.1371/journal.pone.0082368 PMC3869691

[32] Hill MF, Bordoni B. Hyperlipidemia. StatPearls. Treasure Island (FL) ineligible companies. Disclosure: Bruno Bordoni declares no relevant financial relationships with ineligible companies. 2024.

[33] Umeh K. Self-rated health and multimorbidity in patients with type 2 diabetes. J Health Psychol. 2022;27(7):1659–78. 33765898 10.1177/13591053211001419 PMC9092907

[34] Noh JW, Chang Y, Park M, Kwon YD, Ryu S. Self-rated health and the risk of incident type 2 diabetes mellitus: a cohort study. Sci Rep. 2019;9(1):3697. 30842537 10.1038/s41598-019-40090-y PMC6403398

[35] Rottenkolber M, Gar C, Then C, Wanger L, Sacco V, Banning F, et al. A pathophysiology of type 2 diabetes unrelated to metabolic syndrome. J Clin Endocrinol Metab. 2021;106(5):1460–71. 33515032 10.1210/clinem/dgab057 PMC8063234

[36] Hsieh HH, Chang CM, Liu LW, Huang HC. The relative contribution of dietary habits, leisure-time exercise, exercise attitude, and body mass index to self-rated health among college students in Taiwan. Int J Environ Res Public Health. 2018;15(5). 10.3390/ijerph15050967 PMC5982006 29751682

[37] Zarini GG, Vaccaro JA, Canossa Terris MA, Exebio JC, Tokayer L, Antwi J, et al. Lifestyle behaviors and self-rated health: the living for health program. J Environ Public Health. 2014;2014:315042. 25530764 10.1155/2014/315042 PMC4228703

[38] Mindell J, Biddulph JP, Hirani V, Stamatakis E, Craig R, Nunn S, et al. Cohort profile: the health survey for England. Int J Epidemiol. 2012;41(6):1585–93. 22253315 10.1093/ije/dyr199

[39] NHS Digital. Health Survey for England 2019 [NS] - NHS Digital 2022 [cited 2022 30 June]. Available from: https://digital.nhs.uk/data-and-information/publications/statistical/health-survey-for-england/2019.

[40] Taylor BC, Wilt TJ, Welch HG. Impact of diastolic and systolic blood pressure on mortality: implications for the definition of “normal.” J Gen Intern Med. 2011;26(7):685–90. 21404131 10.1007/s11606-011-1660-6 PMC3138604

[41] Cavero-Redondo I, Martinez-Vizcaino V, Alvarez-Bueno C, Agudo-Conde C, Lugones-Sanchez C, Garcia-Ortiz L. Metabolic syndrome including glycated hemoglobin A1c in adults: is it time to change? J Clin Med. 2019;8(12). 10.3390/jcm8122090 PMC6947260 31805696

[42] Sherwani SI, Khan HA, Ekhzaimy A, Masood A, Sakharkar MK. Significance of HbA1c test in diagnosis and prognosis of diabetic patients. Biomark Insights. 2016;11:95–104. 27398023 10.4137/BMI.S38440 PMC4933534

[43] Gerich JE. Insulin resistance is not necessarily an essential component of type 2 diabetes. J Clin Endocrinol Metab. 2000;85(6):2113–5. 10852436 10.1210/jcem.85.6.6646

[44] Nuttall FQ. Body mass index: obesity, BMI, and health: a critical review. Nutr Today. 2015;50(3):117–28. 27340299 10.1097/NT.0000000000000092 PMC4890841

[45] Sun K, Liu J, Ning G. Active smoking and risk of metabolic syndrome: a meta-analysis of prospective studies. PLoS ONE. 2012;7(10):e47791. 23082217 10.1371/journal.pone.0047791 PMC3474781

[46] Sun K, Ren M, Liu D, Wang C, Yang C, Yan L. Alcohol consumption and risk of metabolic syndrome: a meta-analysis of prospective studies. Clin Nutr. 2014;33(4):596–602. 24315622 10.1016/j.clnu.2013.10.003

[47] Slagter SN, van Vliet-Ostaptchouk JV, Vonk JM, Boezen HM, Dullaart RP, Kobold AC, et al. Combined effects of smoking and alcohol on metabolic syndrome: the LifeLines cohort study. PLoS ONE. 2014;9(4):e96406. 24781037 10.1371/journal.pone.0096406 PMC4004580

[48] VanderWeele TJ, Mathur MB. Some desirable properties of the Bonferroni correction: is the Bonferroni correction really so bad? Am J Epidemiol. 2019;188(3):617–8. 30452538 10.1093/aje/kwy250 PMC6395159

[49] Faul F, Erdfelder E, Buchner A, Lang AG. Statistical power analyses using G*Power 3.1: Tests for correlation and regression analyses. Behav Res Methods. 2009;41(4):1149–60. 19897823 10.3758/BRM.41.4.1149

[50] Gelman A, Hill J. Sample size and power calculations. Data analysis using regression and multilevel/hierarchical models (analytical methods for social research). Cambridge: Cambridge University Press; 2006.

[51] Rubin LH, Witkiewitz K, Andre JS, Reilly S. Methods for handling missing data in the behavioral neurosciences: don’t throw the baby rat out with the bath water. J Undergrad Neurosci Educ. 2007;5(2):A71–7. 23493038 PMC3592650

[52] Stavseth MR, Clausen T, Roislien J. How handling missing data may impact conclusions: a comparison of six different imputation methods for categorical questionnaire data. SAGE Open Med. 2019;7:2050312118822912. 30671242 10.1177/2050312118822912 PMC6329020

[53] James M, Varghese TP, Sharma R, Chand S. Association between metabolic syndrome and diabetes mellitus according to international diabetic federation and national cholesterol education program adult treatment panel III criteria: a cross-sectional Study. J Diabetes Metab Disord. 2020;19(1):437–43. 32550195 10.1007/s40200-020-00523-2 PMC7270215

[54] Yoon J, Kim J, Son H. Gender differences of health behaviors in the risk of metabolic syndrome for middle-aged adults: a national cross-sectional study in South Korea. Int J Env Res Pub He. 2021;18(7). 10.3390/ijerph18073699 PMC8037099 33916247

[55] Meloni A, Cadeddu C, Cugusi L, Donataccio MP, Deidda M, Sciomer S, et al. Gender differences and cardiometabolic risk: the importance of the risk factors. Int J Mol Sci. 2023;24(2). 10.3390/ijms24021588 PMC9864423 36675097

[56] Palmer AK, Jensen MD. Metabolic changes in aging humans: current evidence and therapeutic strategies. J Clin Invest. 2022;132(16). 10.1172/JCI158451 PMC9374375 35968789

[57] Hooper D, Coughlan J, Mullen MR. Structural equation modelling: guidelines for determining model fit. Electr J Bus Res Methods. 2008;6(1):53–60.

[58] Memon R, Levitt D, Prado SND, SR, Munir K, Lamos E. Knowledge of hemoglobin A1c and glycemic control in an urban population. Cureus. 2021;13(3):e13995. 33880312 10.7759/cureus.13995 PMC8053309

[59] Gopalan A, Kellom K, McDonough K, Schapira MM. Exploring how patients understand and assess their diabetes control. BMC Endocr Disord. 2018;18(1):79. 30400859 10.1186/s12902-018-0309-4 PMC6219190

[60] Nielsen AB, Gannik D, Siersma V, Olivarius NF. The relationship between HbA1c level, symptoms and self-rated health in type 2 diabetic patients. Scand J Prim Health Care. 2011;29(3):157–64. 21707235 10.3109/02813432.2011.585542 PMC3347958

[61] Kautzky-Willer A, Kosi L, Lin J, Mihaljevic R. Gender-based differences in glycaemic control and hypoglycaemia prevalence in patients with type 2 diabetes: results from patient-level pooled data of six randomized controlled trials. Diabetes Obes Metab. 2015;17(6):533–40. 25678212 10.1111/dom.12449 PMC6680342

[62] Zajacova A, Huzurbazar S, Todd M. Gender and the structure of self-rated health across the adult life span. Soc Sci Med. 2017;187:58–66. 28654822 10.1016/j.socscimed.2017.06.019 PMC5554534

[63] Goldstein KM, Stechuchak KM, Zullig LL, Oddone EZ, Olsen MK, McCant FA, et al. Impact of gender on satisfaction and confidence in cholesterol control among veterans at risk for cardiovascular disease. J Womens Health (Larchmt). 2017;26(7):806–14. 28192012 10.1089/jwh.2016.5739 PMC5507731

[64] Russo G, Pintaudi B, Giorda C, Lucisano G, Nicolucci A, Cristofaro MR, et al. Age- and gender-related differences in LDL-cholesterol management in outpatients with type 2 diabetes mellitus. Int J Endocrinol. 2015;2015:957105. 25873960 10.1155/2015/957105 PMC4383267

[65] Roeters van Lennep JE, Tokgozoglu LS, Badimon L, Dumanski SM, Gulati M, Hess CN, et al. Women, lipids, and atherosclerotic cardiovascular disease: a call to action from the European Atherosclerosis Society. Eur Heart J. 2023;44(39):4157–73. 37611089 10.1093/eurheartj/ehad472 PMC10576616

[66] Havakuk O, Perl ML, Praisler O, Barkagan M, Sadeh B, Margolis G, et al. The awareness to metabolic syndrome among hospital health providers. Diabetes Metab Syndr. 2017;11(3):193–7. 27707551 10.1016/j.dsx.2016.09.005

[67] Wang Q, Chair SY, Wong EM, Taylor-Piliae RE, Qiu XCH, Li XM. Metabolic syndrome knowledge among adults with cardiometabolic risk factors: a cross-sectional study. Int J Environ Res Public Health. 2019;16(1). 10.3390/ijerph16010159 PMC6338970 30626137

[68] Ostan R, Monti D, Gueresi P, Bussolotto M, Franceschi C, Baggio G. Gender, aging and longevity in humans: an update of an intriguing/neglected scenario paving the way to a gender-specific medicine. Clin Sci (Lond). 2016;130(19):1711–25. 27555614 10.1042/CS20160004 PMC4994139

[69] Abad-Diez JM, Calderon-Larranaga A, Poncel-Falco A, Poblador-Plou B, Calderon-Meza JM, Sicras-Mainar A, et al. Age and gender differences in the prevalence and patterns of multimorbidity in the older population. BMC Geriatr. 2014;14:75. 24934411 10.1186/1471-2318-14-75 PMC4070347

